# Supplementary material for: Characterizing human mobility patterns in rural settings of sub-Saharan Africa
Source: eLife. 2021 Sep 17;10:e68441. doi: 10.7554/eLife.68441 (PMC8448534; doi:10.7554/eLife.68441)
Supplement: Figure 2—source data 1. [file elife-68441-fig2-data1.docx]

**Figure 2 - source data file** **1**

| **Gravity models with power distance kernel** | | | | | | | | | | | |
| --- | --- | --- | --- | --- | --- | --- | --- | --- | --- | --- | --- |
| Model | Trip type | γ | | α | | β | | | θ | | DIC |
| **Namibia** | | | | | | | | | | | |
| Basic |  | 1.42 (<0.01) | | 1.17 (<0.01) | | 1.17 (<0.01) | | | 3.06E-05 (2.9E-07) | | 6.12E+06 (2.72) |
| Regional | Intra | 0.73 (<0.01) | | 1.21 (<0.01) | | 1.21 (<0.01) | | | 1.92E-06 (2.0E-08) | | 4.61E+06 (3.96) |
|  | Inter | 1.29 (<0.01) | | 1.25 (<0.01) | | 1.25 (<0.01) | | |  |  |  |
| Urbanicity | Rural - rural | 1.47 (<0.01) | | 0.63 (<0.01) | | 0.63 (<0.01) | | | 1.792 (0.04) | | 4.56E+06 (7.49) |
|  | Rural - urban | 1.95 (<0.01) | | 0.83 (<0.01) | | 0.81 (<0.01) | | |  |  |  |
|  | Urban - rural | 1.95 (<0.01) | | 0.81 (<0.01) | | 0.82 (<0.01) | | |  |  |  |
|  | Urban - urban | 1.07 (<0.01) | | 0.62 (<0.01) | | 0.63 (<0.01) | | |  |  |  |
| Regional & Urbanicity | Intra, rural - rural | 0.83 (<0.01) | | 0.68 (<0.01) | | 0.68 (<0.01) | | | 0.10 (2.95E-3) | | 3.62E+06 (84.37) |
|  | Inter, rural - rural | 1.49 (<0.01) | | 0.75 (<0.01) | | 0.75 (<0.01) | | |  |  |  |
|  | Intra, rural - urban | 1.56 (<0.01) | | 0.97 (<0.01) | | 0.81 (<0.01) | | |  |  |  |
|  | Inter, rural - urban | 1.65 (<0.01) | | 0.75 (<0.01) | | 0.96 (<0.01) | | |  |  |  |
|  | Intra, urban - rural | 1.55 (<0.01) | | 0.82 (<0.01) | | 0.96 (<0.01) | | |  |  |  |
|  | Inter, urban - rural | 1.64 (<0.01) | | 0.96 (<0.01) | | 0.74 (<0.01) | | |  |  |  |
|  | Intra, urban - urban | 5.35 (2.16) | | 1.88 (0.73) | | 1.06 (0.19) | | |  |  |  |
|  | Inter, urban - urban | 1.1 (<0.01) | | 0.75 (<0.01) | | 0.76 (<0.01) | | |  |  |  |
| **Kenya** | | | | | | | | | | | |
| Basic |  | 2.06 (<0.01) | | 1.11 (<0.01) | | 1.11 (<0.01) | | | 1.62E-04 (2.9E-07) | | 3.48E+08 (3.82) |
| Regional | Intra | 1.79 (<0.01) | | 1.12 (<0.01) | | 1.13 (<0.01) | | | 4.89E-05 (2.9E-07) | | 3.40E+08 (134.95) |
|  | Inter | 2.1 (<0.01) | | 1.16 (<0.01) | | 1.16 (<0.01) | | |  |  |  |
| Urbanicity | Rural - rural | 2.7 (<0.01) | | 1.39 (<0.01) | | 1.38 (<0.01) | | | 6.17E-06 (1.17E-08) | | 2.53E+08 (8.09) |
|  | Rural - urban | 3 (<0.01) | | 1.31 (<0.01) | | 1.53 (<0.01) | | |  |  |  |
|  | Urban - rural | 3.01 (<0.01) | | 1.53 (<0.01) | | 1.31 (<0.01) | | |  |  |  |
|  | Urban - urban | 2.17 (<0.01) | | 1.24 (<0.01) | | 1.24 (<0.01) | | |  |  |  |
| Regional & Urbanicity | Intra, rural - rural | 2.14 (<0.01) | | 1.33 (<0.01) | | 1.33 (<0.01) | | | 2.11E-06 (2.44E-08) | | 2.43E+08 (904.7) |
|  | Inter, rural - rural | 3.12 (<0.01) | | 1.51 (<0.01) | | 1.49 (<0.01) | | |  |  |  |
|  | Intra, rural - urban | 2.62 (<0.01) | | 1.33 (<0.01) | | 1.47 (<0.01) | | |  |  |  |
|  | Inter, rural - urban | 3.64 (<0.01) | | 1.64 (<0.01) | | 1.53 (<0.01) | | |  |  |  |
|  | Intra, urban - rural | 2.63 (<0.01) | | 1.48 (<0.01) | | 1.32 (<0.01) | | |  |  |  |
|  | Inter, urban - rural | 3.67 (<0.01) | | 1.53 (<0.01) | | 1.65 (<0.01) | | |  |  |  |
|  | Intra, urban - urban | 2.14 (<0.01) | | 1.28 (<0.01) | | 1.28 (<0.01) | | |  |  |  |
|  | Inter, urban - urban | 2.13 (<0.01) | | 1.27 (<0.01) | | 1.27 (<0.01) | | |  |  |  |
| **Burkina Faso** | | | | | | | | | | | |
| Basic |  | 2.19 (<0.01) | | 0.84 (<0.01) | | 0.84 (<0.01) | | | 1.87E-03 (1.3E-04) | | 2.67E+05 (3.12) |
| Regional | Intra | 1.81 (0.01) | | 0.94 (<0.01) | | 0.94 (<0.01) | | | 4.11E-05 (2.7E-06) | | 2.52E+05 (4.11) |
|  | Inter | 2.04 (<0.01) | | 0.95 (<0.01) | | 0.95 (<0.01) | | |  |  |  |
| Urbanicity | Rural - rural | 3.04 (<0.01) | | 1.23 (<0.01) | | 1.23 (0.01) | | | 4.24E-06 (1.33E-06) | | 2.05E+05 (30.95) |
|  | Rural - urban | 1.17 (0.01) | | 1.17 (0.02) | | 0.64 (0.03) | | |  |  |  |
|  | Urban - rural | 1.18 (0.01) | | 0.63 (0.02) | | 1.18 (0.02) | | |  |  |  |
|  | Urban - urban | 2.09 (2.16) | | 1.99 (2.01) | | 2.04 (2.03) | | |  |  |  |
| Regional & Urbanicity | Intra, rural - rural | 1.73 (0.01) | | 1.07 (<0.01) | | 1.06 (<0.01) | | | 1.17E-06 (3.09E-07) | | 1.93E+05 (18.43) |
|  | Inter, rural - rural | 3.44 (<0.01) | | 1.35 (<0.01) | | 1.35 (0.01) | | |  |  |  |
|  | Intra, rural - urban | 2.05 (2.09) | | 1.98 (2.01) | | 1.96 (1.93) | | |  |  |  |
|  | Inter, rural - urban | 1.18 (0.01) | | 1.18 (0.02) | | 0.72 (0.03) | | |  |  |  |
|  | Intra, urban - rural | 2.07 (2.04) | | 1.98 (1.99) | | 2 (2.06) | | |  |  |  |
|  | Inter, urban - rural | 1.17 (<0.01) | | 0.73 (0.02) | | 1.16 (0.01) | | |  |  |  |
|  | Intra, urban - urban | 2.01 (2.07) | | 2.01 (1.95) | | 2.06 (2.07) | | |  |  |  |
|  | Inter, urban - urban | 1.98 (1.98) | | 1.99 (1.98) | | 1.98 (1.99) | | |  |  |  |
| **Zambia** | | | | | | | | | | | |
| Basic |  | 2.05 (<0.01) | | 0.7 (<0.01) | | 0.7 (<0.01) | | | 0.43 (7.23E-03) | | 2.40E+06 (2.91) |
| Regional | Intra | 1.44 (<0.01) | | 0.72 (<0.01) | | 0.72 (<0.01) | | | 0.02 (4.36E-04) | | 2.10E+06 (3.76) |
|  | Inter | 2.56 (<0.01) | | 0.91 (<0.01) | | 0.91 (<0.01) | | |  |  |  |
| Urbanicity | Rural - rural | 2.15 (<0.01) | | 0.69 (<0.01) | | 0.69 (<0.01) | | | 0.87 (3.61E-20) | | 2.38E+06 (10.28) |
|  | Rural - urban | 1.94 (<0.01) | | 0.53 (<0.01) | | 0.76 (<0.01) | | |  |  |  |
|  | Urban - rural | 1.94 (<0.01) | | 0.76 (<0.01) | | 0.53 (<0.01) | | |  |  |  |
|  | Urban - urban | 2.27 (0.03) | | 0.73 (<0.01) | | 0.73 (<0.01) | | |  |  |  |
| Regional & Urbanicity | Intra, rural - rural | 1.48 (<0.01) | | 0.68 (<0.01) | | 0.68 (<0.01) | | | 0.07 (4.48E-03) | | 2.01E+06 (25.54) |
|  | Inter, rural - rural | 2.7 (<0.01) | | 0.89 (<0.01) | | 0.89 (<0.01) | | |  |  |  |
|  | Intra, rural - urban | 1.41 (<0.01) | | 0.37 (<0.01) | | 0.94 (<0.01) | | |  |  |  |
|  | Inter, rural - urban | 2.91 (<0.01) | | 1.57 (<0.01) | | 0.4 (<0.01) | | |  |  |  |
|  | Intra, urban - rural | 1.41 (<0.01) | | 0.94 (<0.01) | | 0.37 (<0.01) | | |  |  |  |
|  | Inter, urban - rural | 2.91 (<0.01) | | 0.4 (<0.01) | | 1.57 (<0.01) | | |  |  |  |
|  | Intra, urban - urban | 2.04 (2) | | 2.04 (2.01) | | 2.02 (1.95) | | |  |  |  |
|  | Inter, urban - urban | 2.05 (0.04) | | 0.78 (0.01) | | 0.78 (<0.01) | | |  |  |  |
| **Gravity models with exponential distance kernel** | | | | | | | | | | | |
| Model | Trip type | | D | | α | | β | θ | | DIC | |
| **Namibia** | | | | | | | | | | | |
| Basic |  | | 102.83 (0.05) | | 1.18 (<0.01) | | 1.17 (<0.01) | 2.03E-07 (1.8E-09) | | 7.1E+06 (2.8) | |
| Regional | Intra | | 93.26 (0.13) | | 1.24 (<0.01) | | 1.24 (<0.01) | 1.17E-07 (1.1E-09) | | 4.98E+06 (3.7) | |
|  | Inter | | 166.62 (0.14) | | 1.14 (<0.01) | | 1.14 (<0.01) |  |  |  |  |
| Urbanicity | Rural - rural | | 82.87 (0.06) | | 0.85 (<0.01) | | 0.85 (<0.01) | 1.50E-04 (2.11E-06) | | 5.84E+06 (5.23) | |
|  | Rural - urban | | 95.63 (0.08) | | 1.19 (<0.01) | | 0.67 (<0.01) |  |  |  |  |
|  | Urban - rural | | 96.14 (0.08) | | 0.68 (<0.01) | | 1.19 (<0.01) |  |  |  |  |
|  | Urban - urban | | 210.25 (0.76) | | 0.84 (<0.01) | | 0.85 (<0.01) |  |  |  |  |
| Regional & Urbanicity | Intra, rural - rural | | 90.57 (0.14) | | 0.87 (<0.01) | | 0.87 (<0.01) | 1.77E-04 (2.79E-06) | | 4.18E+06 (354.55) | |
|  | Inter, rural - rural | | 115.67 (0.13) | | 0.79 (<0.01) | | 0.79 (<0.01) |  |  |  |  |
|  | Intra, rural - urban | | 51.73 (0.14) | | 1.12 (<0.01) | | 0.8 (<0.01) |  |  |  |  |
|  | Inter, rural - urban | | 181.37 (0.41) | | 0.76 (<0.01) | | 0.86 (<0.01) |  |  |  |  |
|  | Intra, urban - rural | | 51.96 (0.14) | | 0.81 (<0.01) | | 1.11 (<0.01) |  |  |  |  |
|  | Inter, urban - rural | | 184.31 (0.41) | | 0.87 (<0.01) | | 0.75 (<0.01) |  |  |  |  |
|  | Intra, urban - urban | | 1.76 (0.71) | | 2.43 (1.74) | | 1.97 (0.37) |  |  |  |  |
|  | Inter, urban - urban | | 273.98 (1.23) | | 0.82 (<0.01) | | 0.82 (<0.01) |  |  |  |  |
| **Kenya** | | | | | | | | | | | |
| Basic |  | | 47.52 (<0.01) | | 1.12 (<0.01) | | 1.12 (<0.01) | 1.41E-07 (1.8E-10) | | 4.49E+08 (2.6) | |
| Regional | Intra | | 33.31 (<0.01) | | 1.27 (<0.01) | | 1.27 (<0.01) | 5.18E-09 (2.5E-11) | | 4.23E+08 (155.7) | |
|  | Inter | | 56.71 (<0.01) | | 1.23 (<0.01) | | 1.23 (<0.01) |  |  |  |  |
| Urbanicity | Rural - rural | | 50.02 (0.01) | | 1.26 (<0.01) | | 1.25 (<0.01) | 7.32E-09 (1.02E-11) | | 3.96E+08 (6.43) | |
|  | Rural - urban | | 38.53 (<0.01) | | 1.2 (<0.01) | | 1.35 (<0.01) |  |  |  |  |
|  | Urban - rural | | 38.18 (<0.01) | | 1.35 (<0.01) | | 1.2 (<0.01) |  |  |  |  |
|  | Urban - urban | | 42.46 (<0.01) | | 1.23 (<0.01) | | 1.23 (<0.01) |  |  |  |  |
| Regional & Urbanicity | Intra, rural - rural | | 52.11 (0.02) | | 1.38 (<0.01) | | 1.38 (<0.01) | 2.51E-10 (3.59E-12) | | 3.48E+08 (611.8) | |
|  | Inter, rural - rural | | 51.85 (0.02) | | 1.38 (<0.01) | | 1.37 (<0.01) |  |  |  |  |
|  | Intra, rural - urban | | 32.17 (<0.01) | | 1.28 (<0.01) | | 1.55 (<0.01) |  |  |  |  |
|  | Inter, rural - urban | | 41.71 (<0.01) | | 1.36 (<0.01) | | 1.42 (<0.01) |  |  |  |  |
|  | Intra, urban - rural | | 32.09 (<0.01) | | 1.55 (<0.01) | | 1.28 (<0.01) |  |  |  |  |
|  | Inter, urban - rural | | 40.98 (<0.01) | | 1.41 (<0.01) | | 1.37 (<0.01) |  |  |  |  |
|  | Intra, urban - urban | | 19.34 (<0.01) | | 1.41 (<0.01) | | 1.41 (<0.01) |  |  |  |  |
|  | Inter, urban - urban | | 54.48 (<0.01) | | 1.33 (<0.01) | | 1.33 (<0.01) |  |  |  |  |
| **Burkina Faso** | | | | | | | | | | | |
| Basic |  | | 51.82 (0.12) | | 0.85 (<0.01) | | 0.85 (<0.01) | 5.78E-07 (3.4E-08) | | 2.43E+05 (2.9) | |
| Regional | Intra | | 40.18 (0.3) | | 0.99 (<0.01) | | 0.99 (<0.01) | 3.53E-08 (2.2E-09) | | 2.29E+05 (3.6) | |
|  | Inter | | 58.49 (0.17) | | 0.94 (<0.01) | | 0.94 (<0.01) |  |  |  |  |
| Urbanicity | Rural - rural | | 34.37 (0.09) | | 1.15 (0.01) | | 1.15 (0.01) | 7.50E-10 (1.93E-10) | | 1.87E+05 (46.24) | |
|  | Rural - urban | | 115.66 (1.14) | | 1.11 (0.02) | | 0.98 (0.04) |  |  |  |  |
|  | Urban - rural | | 114.83 (1.11) | | 0.98 (0.03) | | 1.11 (0.01) |  |  |  |  |
|  | Urban - urban | | 2.02 (2) | | 2.03 (2.06) | | 2.02 (2.02) |  |  |  |  |
| Regional & Urbanicity | Intra, rural - rural | | 40.69 (0.32) | | 1.14 (<0.01) | | 1.14 (<0.01) | 7.35E-10 (1.52E-10) | | 1.86E+05 (16.14) | |
|  | Inter, rural - rural | | 34.12 (0.11) | | 1.15 (<0.01) | | 1.15 (<0.01) |  |  |  |  |
|  | Intra, rural - urban | | 2.03 (2.01) | | 2.03 (2.07) | | 1.94 (1.93) |  |  |  |  |
|  | Inter, rural - urban | | 115.53 (1.12) | | 1.11 (0.02) | | 0.97 (0.03) |  |  |  |  |
|  | Intra, urban - rural | | 2.05 (2.09) | | 2.01 (1.99) | | 2 (1.98) |  |  |  |  |
|  | Inter, urban - rural | | 114.85 (1.09) | | 0.98 (0.02) | | 1.11 (0.02) |  |  |  |  |
|  | Intra, urban - urban | | 2 (2.03) | | 1.98 (2.01) | | 1.95 (1.95) |  |  |  |  |
|  | Inter, urban - urban | | 1.93 (1.91) | | 2 (2.04) | | 1.97 (1.96) |  |  |  |  |
| **Zambia** | | | | | | | | | | | |
| Basic |  | | 56.15 (0.04) | | 0.81 (<0.01) | | 0.81 (<0.01) | 2.37E-05 (3.50E-07) | | 2.172E+06 (2.98) | |
| Regional | Intra | | 57.95 (0.09) | | 0.84 (<0.01) | | 0.84 (<0.01) | 1.21E-05 (2.18E-07) | | 2.08E+06 (4.2) | |
|  | Inter | | 69.94 (0.08) | | 0.8 (<0.01) | | 0.8 (<0.01) |  |  |  |  |
| Urbanicity | Rural - rural | | 53.9 (0.05) | | 0.78 (<0.01) | | 0.78 (<0.01) | 5.30E-05 (1.21E-06) | | 2.16E+06 (5.22) | |
|  | Rural - urban | | 58.63 (0.09) | | 0.81 (<0.01) | | 0.75 (<0.01) |  |  |  |  |
|  | Urban - rural | | 58.63 (0.09) | | 0.75 (<0.01) | | 0.81 (<0.01) |  |  |  |  |
|  | Urban - urban | | 83.28 (1.45) | | 0.75 (<0.01) | | 0.75 (<0.01) |  |  |  |  |
| Regional & Urbanicity | Intra, rural - rural | | 53.4 (0.08) | | 0.78 (<0.01) | | 0.78 (<0.01) | 5.85E-05 (1.36E-06) | | 2.02E+06 (7.38) | |
|  | Inter, rural - rural | | 66.52 (0.11) | | 0.74 (<0.01) | | 0.74 (<0.01) |  |  |  |  |
|  | Intra, rural - urban | | 59.06 (0.24) | | 0.53 (<0.01) | | 0.99 (<0.01) |  |  |  |  |
|  | Inter, rural - urban | | 69.64 (0.19) | | 1.11 (<0.01) | | 0.45 (<0.01) |  |  |  |  |
|  | Intra, urban - rural | | 59.05 (0.24) | | 0.99 (<0.01) | | 0.53 (<0.01) |  |  |  |  |
|  | Inter, urban - rural | | 69.62 (0.19) | | 0.45 (<0.01) | | 1.11 (<0.01) |  |  |  |  |
|  | Intra, urban - urban | | 2.03 (2.05) | | 2 (1.98) | | 2 (1.98) |  |  |  |  |
|  | Inter, urban - urban | | 83.28 (1.48) | | 0.74 (<0.01) | | 0.74 (<0.01) |  |  |  |  |
| **Radiation Model** | | | | | | | | | | | |
| Country |  | | σ | |  | |  |  | | DIC | |
| Namibia |  | | 6.93 (3.43E-03) | |  | |  |  | | 8.68E+06 (1.28) | |
| Kenya |  | | 37.89 (1.92E-03) | |  | |  |  | | 4.26E+08 (1.06) | |
| Burkina Faso |  | | 0.042 (1.03E-4) | |  | |  |  | | 3.39E+05 (1.64) | |
| Zambia |  | | 0.53 (4.10E-04) | |  | |  |  | | 4.32E+06 (1.39) | |
