# Supplementary material for: Characterizing human mobility patterns in rural settings of sub-Saharan Africa
Source: eLife. 2021 Sep 17;10:e68441. doi: 10.7554/eLife.68441 (PMC8448534; doi:10.7554/eLife.68441)
Supplement: Supplementary file 1. — A. Basic characteristics of countries and trips. B. Key of region and district IDs and names. See Figure 1—figure supplement 9 for a map. [file elife-68441-supp1.docx]

**Supplementary file 1A**. Basic characteristics of countries and trips

|  | **Namibia** | **Kenya** | **Burkina Faso** | **Zambia** |
| --- | --- | --- | --- | --- |
| **Call data record (CDR) details** | | | |  |
| Mobile phone operator |  | Safaricom | Telecel Faso (Moov) | Zamtel |
| CDR time window | 10/02/2010 –04/30/2014 | 06/01/2008 – 07/03/2009  (excludes 02/2009) | 01/01/2016 – 12/31/2016 | 08/01/2020 – 12/30/2020 |
| Number of records | 1,844,566,007 | 84,812,816,520 | 37,693,904 | 112,049,422 |
| Proportion of operator’s subscribers | 100% | 100% | 1.4% | 100% |
| **Country details at time of CDR collection** | | | |  |
| WorldPop File | nam_ppp_v2b_2010 | ken_ppp_2009 | bfa_ppp_2016 |  |
| Population size | 2,079,341 | 38,610,097 | 19,705,174 | 18,383,956 |
| Administrative 1 units | 13 Regions | 8 Provinces | 13 Regions | 10 Provinces |
| Administrative 2 units | 96 Constituencies* | 69 Counties | 45 Provinces | 116 Districts |
| **Trip type details** | | | |  |
| Rural adm2 units | 90 (93.75%) | 30 (43.5%) | 44 (97.8%) | 112 (96.6%) |
| Urban adm2 units | 6 (6.25%) | 39 (56.5%) | 1 (2.2%) | 4 (3.4%) |
| **Total possible routes** | **9216** | **4761** | **2025** | **13456** |
| Regional |  |  |  |  |
| Intra-regional | 698 (7.6%) | 740 (15.5%) | 130 (6.4%) | 1288 (9.6%) |
| Inter-regional | 8422 (91.4%) | 3952 (83.0%) | 1850 (91.4%) | 12052 (89.6%) |
| Urbanicity |  |  |  |  |
| Rural – Rural routes | 8010 (86.9%) | 870 (18.3%) | 1892 (93.4%) | 12432 (92.4%) |
| Rural $\leftrightarrow$ Urban routes | 540 (5.9%) | 1170 (24.6%) | 44 (2.2%) | 448 (3.33%) |
| Urban – Urban routes | 30 (0.3%) | 1482 (31.1%) | 0 (0%) | 12 (0.1%) |
| Regional & Urbanicity |  |  |  |  |
| Intra: Rural – Rural | 642 (7.0%) | 204 (4.3%) | 130 (6.4%) | 1214 (9.0%) |
| Inter: Rural – Rural | 7368 (70.0%) | 666 (14.0%) | 1762 (87.0%) | 11218 (83.4%) |
| Intra: Rural $\leftrightarrow$ Urban | 27 (0.3%) | 143 (3.0%) | 0 (0%) | 37 (0.3%) |
| Inter: Rural$\leftrightarrow$ Urban | 513 (5.6%) | 1027 (21.6%) | 44 (2.2%) | 411 (3.1%) |
| Intra: Urban - Urban | 2 (0.02%) | 250 (5.3%) | 0 (0%) | 0 (0%) |
| Inter: Urban - Urban | 28 (0.3%) | 1232 (25.9%) | 0 (0%) | 12 (0.1%) |
| **Total monthly trips (avg (%))** | **3,786,819** | **331,930,406** | **176,560** | **1,632,094** |
| Regional |  |  |  |  |
| Intra-regional | 2364052 (62.4%) | 148071459 (44.6%) | 52785 (29.9%) | 1179326 (72.3%) |
| Inter-regional | 1422767 (37.6%) | 183858947 (55.4%) | 123775 (70.1%) | 452768 (27.7%) |
| Urbanicity |  |  |  |  |
| Rural – Rural trips | 2332523 (61.6%) | 12446805 (3.8%) | 124553 (70.5%) | 871138 (53.4%) |
| Rural – Urban trips | 637896 (16.9%) | 43751899 (13.2%) | 25969 (14.7%) | 376543 (23.1%) |
| Urban – Rural trips | 640704 (16.9%) | 43711713 (13.2%) | 26038 (14.8%) | 376543 (23.1%) |
| Urban – Urban trips | 175696 (4.6%) | 232019989 (69.9%) | 0 (0%) | 7870 (0.5%) |
| Regional & Urbanicity |  |  |  |  |
| Intra: Rural-to-rural | 1503707 (39.7%) | 9179830 (2.8%) | 52785 (29.9%) | 648218 (39.7%) |
| Inter: Rural-to-rural | 828816 (21.9%) | 3266975 (1.0%) | 71768 (40.7%) | 222920 (13.7%) |
| Intra: Rural-to-urban | 407851 (10.8%) | 20996049 (6.3%) | 0 (0%) | 265554 (16.3%) |
| Inter: Rural-to-urban | 230045 (6.1%) | 22755850 (6.9%) | 25969 (14.7%) | 110989 (6.8%) |
| Intra: Urban-to-rural | 409186 (10.8%) | 20978357 (6.3%) | 0 (0%) | 265554 (16.3%) |
| Inter: Urban-to-rural | 231518 (6.1%) | 22733356 (6.9%) | 26038 (14.8%%) | 110989 (6.8%) |
| Intra: Urban-to-urban | 43308 (1.1%) | 96917223 (29.2%) | 0 (0%) | 0 (0%) |
| Inter: Urban-to-urban | 132388 (3.5%) | 135102766 (40.7%) | 0 (0%) | 7870 (0.5%) |
| **Trip distance (km, avg (SD))** | **483.9 (307.3)** | **316.7 (197.9)** | **269.8 (146.6)** | **381.0 (216.6)** |
| Regional |  |  |  |  |
| Intra-regional | 115.2 (81.1) | 157.1 (119.5) | 101.0 (41.0) | 160.1 (88.6) |
| Inter-regional | 520.4 (295.7) | 352.1 (191.6) | 288.2 (138.9) | 431.9 (200.9) |
| Urbanicity |  |  |  |  |
| Rural – Rural trips | 477.5 (309.3) | 403.0 (191.4) | 279.5 (143.2) | 383.5 (212.2) |
| Rural $\leftrightarrow$ Urban trips | 569.9 (253.1) | 363.0 (193.5) | 200.5 (99.2) | 420.1 (212.2) |
| Urban – Urban trips | 581.1 (375.2) | 207.8 (144.6) | -- | 417.8 (160.7) |
| Regional & Urbanicity |  |  |  |  |
| Intra: Rural-to-rural | 115.5 (82.7) | 239.2 (132.4) | 101.0 (41.0) | 159.9 (86.5) |
| Inter: Rural-to-rural | 509.3 (301.6) | 453.2 (178.2) | 292.6 (139.2) | 429.3 (201.0) |
| Intra: Rural $\leftrightarrow$ Urban | 114.9 (62.8) | 173.8 (108.3) | -- | 162.3 (113.3) |
| Inter: Rural$\leftrightarrow$ Urban | 594.4 (235.9 | 389.4 (188.1) | 200.5 (99.2) | 450.9 (200.0) |
| Intra: Urban-to-urban | 38.0 (0) | 71.1 (37.6) | -- | -- |
| Inter: Urban-to-urban | 619.9 (357.4) | 235.5 (142.6) | -- | 417.8 (160.7) |

**The original map file of Namibia had four areas that were split into constituencies, thus dividing the number of trips being made to a certain area. To address this, we joined Rehobeth East and Rehobeth West to make Rehobeth Urban; Rundu Rural East and Rundu Rural West to make Rundu Rural; Katutura Central, Katutura East, Khomasdal North, Moses Garoeb, Soweto, Tobias Hainyeko, Wanaheda, Windhoek East, and Windhoek West to make Windhoek Urban; and Oshakati East and Oshakati West to make Oshakati Urban.*

**Supplementary file 1B.** Key of region and district IDs and names. **See Figure 1 – figure supplement 9** for a map.

| Region ID | Region Name | District ID | District Name |
| --- | --- | --- | --- |
| Namibia |  |  |  |
| 1 | Zambezi | 1 | Kabe |
| 1 | Zambezi | 2 | Katima Muliro Rural |
| 1 | Zambezi | 3 | Katima Muliro Urban |
| 1 | Zambezi | 4 | Kongola |
| 1 | Zambezi | 5 | Linyandi |
| 1 | Zambezi | 6 | Sibinda |
| 2 | Erongo | 7 | Arandis |
| 2 | Erongo | 8 | Daures |
| 2 | Erongo | 9 | Karibib |
| 2 | Erongo | 10 | Omaruru |
| 2 | Erongo | 11 | Swakopmund |
| 2 | Erongo | 12 | Walvisbay Rural |
| 2 | Erongo | 13 | Walvisbay Urban |
| 3 | Hardap | 14 | Gibeon |
| 3 | Hardap | 15 | Mariental Rural |
| 3 | Hardap | 16 | Mariental Urban |
| 3 | Hardap | 17 | Rehoboth Urban |
| 3 | Hardap | 18 | Rehoboth Rural |
| 4 | Karas | 20 | Berseba |
| 4 | Karas | 21 | Karas |
| 4 | Karas | 22 | Keetmanshoop Rural |
| 4 | Karas | 23 | Keetmanshoop Urban |
| 4 | Karas | 24 | Luderitz |
| 4 | Karas | 25 | Oranjemund |
| 5 | Kavango | 26 | Kahenge |
| 5 | Kavango | 27 | Kapako |
| 5 | Kavango | 28 | Mashare |
| 5 | Kavango | 29 | Mpungu |
| 5 | Kavango | 30 | Mukwe |
| 5 | Kavango | 31 | Ndiyona |
| 5 | Kavango | 32 | Rundu Rural |
| 5 | Kavango | 34 | Rundu Urban |
| 6 | Khomas | 35 | Windhoek Urban |
| 6 | Khomas | 43 | Windhoek Rural |
| 7 | Kunene | 45 | Epupa |
| 7 | Kunene | 46 | Kamanjab |
| 7 | Kunene | 47 | Khorixas |
| 7 | Kunene | 48 | Opuwo |
| 7 | Kunene | 49 | Outjo |
| 7 | Kunene | 50 | Sesfontein |
| 8 | Ohangwena | 51 | Eenhana |
| 8 | Ohangwena | 52 | Endola |
| 8 | Ohangwena | 53 | Engela |
| 8 | Ohangwena | 54 | Epembe |
| 8 | Ohangwena | 55 | Ohangwena |
| 8 | Ohangwena | 56 | Okongo |
| 8 | Ohangwena | 57 | Omulonga |
| 8 | Ohangwena | 58 | Omundaungilo |
| 8 | Ohangwena | 59 | Ondobe |
| 8 | Ohangwena | 60 | Ongenga |
| 8 | Ohangwena | 61 | Oshikango |
| 9 | Omaheke | 62 | Aminius |
| 9 | Omaheke | 63 | Epukiro |
| 9 | Omaheke | 64 | Gobabis |
| 9 | Omaheke | 65 | Kalahari |
| 9 | Omaheke | 66 | Otjinene |
| 9 | Omaheke | 67 | Otjombinde |
| 9 | Omaheke | 68 | Steinhausen |
| 10 | Omusati | 69 | Anamulenge |
| 10 | Omusati | 70 | Elim |
| 10 | Omusati | 71 | Etayi |
| 10 | Omusati | 72 | Ogongo |
| 10 | Omusati | 73 | Okahao |
| 10 | Omusati | 74 | Okalongo |
| 10 | Omusati | 75 | Onesi |
| 10 | Omusati | 76 | Oshikuku |
| 10 | Omusati | 77 | Otamanzi |
| 10 | Omusati | 78 | Outapi |
| 10 | Omusati | 79 | Ruacana |
| 10 | Omusati | 80 | Tsandi |
| 11 | Oshana | 81 | Okaku |
| 11 | Oshana | 83 | Okatyali |
| 11 | Oshana | 85 | Ondangwa |
| 11 | Oshana | 86 | Ongwediva |
| 11 | Oshana | 87 | Oshakati |
| 11 | Oshana | 89 | Uukwiyu |
| 11 | Oshana | 90 | Uuvudhiya |
| 12 | Oshikoto | 91 | Engodi |
| 12 | Oshikoto | 92 | Guinas |
| 12 | Oshikoto | 93 | Okankolo |
| 12 | Oshikoto | 94 | Olukonda |
| 12 | Oshikoto | 95 | Omuntele |
| 12 | Oshikoto | 96 | Omuthiyagwipundi |
| 12 | Oshikoto | 97 | Onayena |
| 12 | Oshikoto | 98 | Oniipa |
| 12 | Oshikoto | 99 | Onyaanya |
| 12 | Oshikoto | 100 | Tsumeb |
| 13 | Otjozondjupa | 101 | Grootfontein |
| 13 | Otjozondjupa | 102 | Okahandja |
| 13 | Otjozondjupa | 103 | Okakarara |
| 13 | Otjozondjupa | 104 | Omatako |
| 13 | Otjozondjupa | 105 | Otavi |
| 13 | Otjozondjupa | 106 | Otjiwarongo |
| 13 | Otjozondjupa | 107 | Tsumkwe |
| Kenya |  |  |  |
| 1 | Central | 1 | Kiambu |
| 1 | Central | 2 | Kirinyaga |
| 1 | Central | 3 | Maragua |
| 1 | Central | 4 | Muranga |
| 1 | Central | 5 | Nyandarua |
| 1 | Central | 6 | Nyeri |
| 1 | Central | 7 | Thika |
| 2 | Coast | 8 | Kilifi |
| 2 | Coast | 9 | Kwale |
| 2 | Coast | 10 | Lamu |
| 2 | Coast | 11 | Malindi |
| 2 | Coast | 12 | Mombasa |
| 2 | Coast | 13 | Taita Taveta |
| 2 | Coast | 14 | Tana River |
| 3 | Eastern | 15 | Embu |
| 3 | Eastern | 16 | Isiolo |
| 3 | Eastern | 17 | Kitui |
| 3 | Eastern | 18 | Machakos |
| 3 | Eastern | 19 | Makueni |
| 3 | Eastern | 20 | Marsabit |
| 3 | Eastern | 21 | Mbeere |
| 3 | Eastern | 22 | Meru Central |
| 3 | Eastern | 23 | Meru North |
| 3 | Eastern | 24 | Meru South |
| 3 | Eastern | 25 | Moyale |
| 3 | Eastern | 26 | Mwingi |
| 3 | Eastern | 27 | Tharaka |
| 4 | Nairobi | 28 | Nairobi |
| 5 | North Eastern | 29 | Garissa |
| 5 | North Eastern | 30 | Mandera |
| 5 | North Eastern | 31 | Wajir |
| 6 | Nyanza | 32 | Bondo |
| 6 | Nyanza | 33 | Central Kisii |
| 6 | Nyanza | 34 | Gucha |
| 6 | Nyanza | 35 | Homa Bay |
| 6 | Nyanza | 36 | Kisumu |
| 6 | Nyanza | 37 | Kuria |
| 6 | Nyanza | 38 | Migori |
| 6 | Nyanza | 39 | Nyamira |
| 6 | Nyanza | 40 | Nyando |
| 6 | Nyanza | 41 | Rachuonyo |
| 6 | Nyanza | 42 | Siaya |
| 6 | Nyanza | 43 | Suba |
| 7 | Rift Valley | 44 | Baringo |
| 7 | Rift Valley | 45 | Bomet |
| 7 | Rift Valley | 46 | Buret |
| 7 | Rift Valley | 47 | Kajiado |
| 7 | Rift Valley | 48 | Keiyo |
| 7 | Rift Valley | 49 | Kericho |
| 7 | Rift Valley | 50 | Koibatek |
| 7 | Rift Valley | 51 | Laikipia |
| 7 | Rift Valley | 52 | Marakwet |
| 7 | Rift Valley | 53 | Nakuru |
| 7 | Rift Valley | 54 | Narok |
| 7 | Rift Valley | 55 | Samburu |
| 7 | Rift Valley | 56 | Trans mara |
| 7 | Rift Valley | 57 | Trans nzoia |
| 7 | Rift Valley | 58 | Turkana |
| 7 | Rift Valley | 59 | Uasin gishu |
| 7 | Rift Valley | 60 | West pokot |
| 8 | Western | 61 | Bungoma |
| 8 | Western | 62 | Busia |
| 8 | Western | 63 | Butere/Mumias |
| 8 | Western | 64 | Kakamega |
| 8 | Western | 65 | Lugari |
| 8 | Western | 66 | Mt Elgon |
| 8 | Western | 67 | Teso |
| 8 | Western | 68 | Vihiga |
| 7 | Rift Valley | 69 | Nandi |
| Burkina Faso | |  |  |
| 1 | Boucle Du Mouhoun | 1 | Bale |
| 1 | Boucle Du Mouhoun | 2 | Banwa |
| 1 | Boucle Du Mouhoun | 3 | Kossi |
| 1 | Boucle Du Mouhoun | 4 | Mouhoun |
| 1 | Boucle Du Mouhoun | 5 | Nayala |
| 1 | Boucle Du Mouhoun | 6 | Sourou |
| 2 | Cascades | 7 | Comoe |
| 2 | Cascades | 8 | Leraba |
| 7 | Centre | 22 | Kadiogo |
| 3 | Centre-est | 9 | Boulgou |
| 3 | Centre-est | 10 | Koulpelogo |
| 3 | Centre-est | 11 | Kouritenga |
| 4 | Centre-nord | 12 | Bam |
| 4 | Centre-nord | 13 | Namentenga |
| 4 | Centre-nord | 14 | Sanmatenga |
| 5 | Centre-ouest | 15 | Boulkiemde |
| 5 | Centre-ouest | 16 | Sanguie |
| 5 | Centre-ouest | 17 | Sissili |
| 5 | Centre-ouest | 18 | Ziro |
| 6 | Centre-sud | 19 | Bazega |
| 6 | Centre-sud | 20 | Nahouri |
| 6 | Centre-sud | 21 | Zoundweogo |
| 8 | Est | 23 | Gnagna |
| 8 | Est | 24 | Gourma |
| 8 | Est | 25 | Komandjoari |
| 8 | Est | 26 | Kompienga |
| 8 | Est | 27 | Tapoa |
| 9 | Haut-bassins | 28 | Houet |
| 9 | Haut-bassins | 29 | Kenedougou |
| 9 | Haut-bassins | 30 | Tuy |
| 10 | Nord | 31 | Loroum |
| 10 | Nord | 32 | Passore |
| 10 | Nord | 33 | Yatenga |
| 10 | Nord | 34 | Zondoma |
| 11 | Plateau-central | 35 | Ganzourgou |
| 11 | Plateau-central | 36 | Kourweogo |
| 11 | Plateau-central | 37 | Oubritenga |
| 12 | Sahel | 38 | Oudalan |
| 12 | Sahel | 39 | Seno |
| 12 | Sahel | 40 | Soum |
| 12 | Sahel | 41 | Yagha |
| 13 | Sud-ouest | 42 | Bougouriba |
| 13 | Sud-ouest | 43 | Ioba |
| 13 | Sud-ouest | 44 | Noumbiel |
| 13 | Sud-ouest | 45 | Poni |
| Zambia | | | |
| 1 | Central | 1 | Chibombo |
| 1 | Central | 2 | Chisamba |
| 1 | Central | 3 | Chitambo |
| 1 | Central | 4 | Itezhi-Tezhi |
| 1 | Central | 5 | Kabwe |
| 1 | Central | 6 | Kapiri Mposhi |
| 1 | Central | 7 | Luano |
| 1 | Central | 8 | Mkushi |
| 1 | Central | 9 | Mumbwa |
| 1 | Central | 10 | Ngabwe |
| 1 | Central | 11 | Serenje |
| 1 | Central | 12 | Shibuyunji |
| 2 | Copperbelt | 13 | Chililabombwe |
| 2 | Copperbelt | 14 | Chingola |
| 2 | Copperbelt | 15 | Kalulushi |
| 2 | Copperbelt | 16 | Kitwe |
| 2 | Copperbelt | 17 | Luanshya |
| 2 | Copperbelt | 18 | Lufwanyama |
| 2 | Copperbelt | 19 | Masaiti |
| 2 | Copperbelt | 20 | Mpongwe |
| 2 | Copperbelt | 21 | Mufulira |
| 2 | Copperbelt | 22 | Ndola |
| 3 | Eastern | 23 | Chadiza |
| 3 | Eastern | 24 | Chipata |
| 3 | Eastern | 25 | Katete |
| 3 | Eastern | 26 | Lundazi |
| 3 | Eastern | 27 | Mambwe |
| 3 | Eastern | 28 | Nyimba |
| 3 | Eastern | 29 | Petauke |
| 3 | Eastern | 30 | Sinda |
| 3 | Eastern | 31 | Vubwi |
| 3 | Eastern | 32 | Chasefu |
| 3 | Eastern | 33 | Chipangali |
| 3 | Eastern | 34 | Kasenengwa |
| 3 | Eastern | 35 | Lumezi |
| 3 | Eastern | 36 | Lusangazi |
| 4 | Luapula | 37 | Chembe |
| 4 | Luapula | 38 | Chiengi |
| 4 | Luapula | 39 | Chipili |
| 4 | Luapula | 40 | Kawambwa |
| 4 | Luapula | 41 | Lunga |
| 4 | Luapula | 42 | Mansa |
| 4 | Luapula | 43 | Milengi |
| 4 | Luapula | 44 | Mwansabombwe |
| 4 | Luapula | 45 | Mwense |
| 4 | Luapula | 46 | Nchelenge |
| 4 | Luapula | 47 | Samfya |
| 4 | Luapula | 48 | Chifunabuli |
| 5 | Lusaka | 49 | Chilanga |
| 5 | Lusaka | 50 | Chirundu |
| 5 | Lusaka | 51 | Chongwe |
| 5 | Lusaka | 52 | Kafue |
| 5 | Lusaka | 53 | Luangwa |
| 5 | Lusaka | 54 | Lusaka |
| 5 | Lusaka | 55 | Rufunsa |
| 6 | Muchinga | 56 | Chama |
| 6 | Muchinga | 57 | Chinsali |
| 6 | Muchinga | 58 | Isoka |
| 6 | Muchinga | 59 | Mafinga |
| 6 | Muchinga | 60 | Mpika |
| 6 | Muchinga | 61 | Nakonde |
| 6 | Muchinga | 62 | Shiwang'Andu |
| 6 | Muchinga | 63 | Kanchibiya |
| 6 | Muchinga | 64 | Lavushimanda |
| 7 | Northern | 65 | Chilubi |
| 7 | Northern | 66 | Kaputa |
| 7 | Northern | 67 | Kasama |
| 7 | Northern | 68 | Luwingu |
| 7 | Northern | 69 | Mbala |
| 7 | Northern | 70 | Mporokoso |
| 7 | Northern | 71 | Mpulungu |
| 7 | Northern | 72 | Mungwi |
| 7 | Northern | 73 | Nsama |
| 7 | Northern | 74 | Senga Hill |
| 7 | Northern | 75 | Lunte |
| 7 | Northern | 76 | Lupososhi |
| 8 | North-Western | 77 | Chavuma |
| 8 | North-Western | 78 | Ikelenge |
| 8 | North-Western | 79 | Kabompo |
| 8 | North-Western | 80 | Kasempa |
| 8 | North-Western | 81 | Manyinga |
| 8 | North-Western | 82 | Mufumbwe |
| 8 | North-Western | 83 | Mwinilunga |
| 8 | North-Western | 84 | Solwezi |
| 8 | North-Western | 85 | Zambezi |
| 8 | North-Western | 86 | Kalumbila |
| 8 | North-Western | 87 | Mushindano |
| 9 | Southern | 88 | Chikankanta |
| 9 | Southern | 89 | Choma |
| 9 | Southern | 90 | Gwembe |
| 9 | Southern | 91 | Kalomo |
| 9 | Southern | 92 | Kazungula |
| 9 | Southern | 93 | Livingstone |
| 9 | Southern | 94 | Mazabuka |
| 9 | Southern | 95 | Monze |
| 9 | Southern | 96 | Namwala |
| 9 | Southern | 97 | Pemba |
| 9 | Southern | 98 | Siavonga |
| 9 | Southern | 99 | Sinazongwe |
| 9 | Southern | 100 | Zimba |
| 10 | Western | 101 | Kalabo |
| 10 | Western | 102 | Kaoma |
| 10 | Western | 103 | Limulunga |
| 10 | Western | 104 | Luampa |
| 10 | Western | 105 | Lukulu |
| 10 | Western | 106 | Mitete |
| 10 | Western | 107 | Mongu |
| 10 | Western | 108 | Mulobezi |
| 10 | Western | 109 | Mwandi |
| 10 | Western | 110 | Nalolo |
| 10 | Western | 111 | Nkeyema |
| 10 | Western | 112 | Senanga |
| 10 | Western | 113 | Sesheke |
| 10 | Western | 114 | Shangombo |
| 10 | Western | 115 | Sikongo |
| 10 | Western | 116 | Sioma |
