# Supplementary material for: Characterizing human mobility patterns in rural settings of sub-Saharan Africa
Source: eLife. 2021 Sep 17;10:e68441. doi: 10.7554/eLife.68441 (PMC8448534; doi:10.7554/eLife.68441)
Supplement: Supplementary file 2. — A. Gravity model variations and radiation model, ranked for each country based on Deviance Information Criterion (DIC) (Standard Deviation) and percent change (%Δ) from basic model. A similar trend in ranking models by best fit was seen for gravity models with power or exponential distance kernel. B. Model fits ranked by Deviance Information Criterion (DIC) for each country. Although the different definitions of urbanicity impacted the distribution of urbanicity trip types, the overall ranking of the model fits was not affected. Generally, the models that used the lower urban threshold (10% urban grid cells) had larger DICs (worse fits) than the models that used the higher urban threshold (50% urban grid cells). The gravity models with the power distance kernel were used here. C. Model fits ranked by Deviance Information Criterion (DIC) for each country at administrative levels 1–3 (when available). Although the different administrative unit boundaries impacted the size of the DIC, the overall ranking of the model fits was not affected. Generally, the models that the larger administrative units (administrative one units) had smaller DICs (better fits) than the models that used the smaller administrative units (administrative three units). [file elife-68441-supp2.docx]

**Supplementary file 2: Model fit comparisons**

**Supplementary file 2A.** Gravity model variations and radiation model, ranked for each country based on Deviance Information Criterion (DIC) (Standard Deviation) and percent change (%Δ) from basic model. A similar trend in ranking models by best fit was seen for gravity models with power or exponential distance kernel.

|  | Namibia | | | Kenya | | | Burkina Faso | | | | Zambia | | |  |
| --- | --- | --- | --- | --- | --- | --- | --- | --- | --- | --- | --- | --- | --- | --- |
| rank | Model | DIC (SD) | %Δ | Model | DIC (SD) | %Δ | Model | DIC (SD) | %Δ | Model | | DIC (SD) | %Δ | Distance function |
| 1 | Regional & Urbanicity | 3.62E+06 (84.4) | 41.0 | Regional & Urbanicity | 2.43E+08 (904.7) | 30.1 | Regional & Urbanicity | 1.93E+05 (18.4) | 27.7 | Regional & Urbanicity | | 2.01E+06 (25.5) | 16.3 | power |
| 2 | Urbanicity | 4.56E+06 (7.5) | 25.6 | Urbanicity | 2.53E+08 (8.1) | 27.2 | Urbanicity | 2.05E+05 (31.0) | 23.1 | Regional | | 2.08E+06 (4.2) | 13.4 | power |
| 3 | Regional | 4.61E+06 (4.0) | 24.8 | Regional | 3.40E+08 (135.0) | 2.1 | Regional | 2.52E+05 (4.1) | 5.7 | Urbanicity | | 2.38E+06 (10.3) | 1.1 | power |
| 4 | Basic | 6.12E+06 (2.7) | 0.0 | Basic | 3.48E+08 (3.82) | 0.0 | Basic | 2.67E+05 (3.1) | 0 | Basic | | 2.40E+06 (2.9) | 0 | power |
| 6 | Radiation | 8.68E+06 (1.3) | -41.7 | Radiation | 4.26E+08 (1.06) | -22.4 | Radiation | 3.39E+05 (1.64) | -27.2 | Radiation | | 4.32E+06 (1.4) | -79.9 | NA |
|  | Namibia | | | Kenya | | | Burkina Faso | | | | Zambia | | |  |
| 1 | Regional & Urbanicity | 4.18E+06 (354.6) | 41.1 | Regional & Urbanicity | 3.48E+08 (611.80) | 22.5 | Regional & Urbanicity | 1.86E+05 (16.1) | 23.5 | Regional & Urbanicity | | 2.02E+06 (7.4) | 7.1 | exponential |
| 2 | Regional | 4.98E+06 (3.7) | 29.8 | Urbanicity | 3.96E+08 (6.43) | 11.8 | Urbanicity | 1.87E+05 (46.2) | 23.1 | Regional | | 2.10E+06 (3.8) | 3.2 | exponential |
| 3 | Urbanicity | 5.84E+06 (5.2) | 17.7 | Regional | 4.23E+08 (155.66) | 5.7 | Regional | 2.29E+05 (3.6) | 5.7 | Urbanicity | | 2.16E+06 (5.2) | 0.5 | exponential |
| 4 | Basic | 7.10E+06 (2.8) | 0.0 | Radiation | 4.26E+08 (1.06) | 5.1 | Basic | 2.43E+05 (2.9) | 0.0 | Basic | | 2.17E+06 (2.98) | 0 | exponential |
| 6 | Radiation | 8.68E+06 (1.3) | -22.2 | Basic | 4.49E+08 (2.58) | 0.0 | Radiation | 3.39E+05 (1.64) | -39.4 | Radiation | | 4.32E+06 (1.4) | -98.9 | NA |

**Supplementary file 2B.** Model fits ranked by Deviance Information Criterion (DIC) (Standard Deviation) for each country at administrative levels 1 – 3 (when available). Although the different administrative unit boundaries impacted the size of the DIC, the overall ranking of the model fits was not affected. Generally, the models that the larger administrative units (administrative 1 units) had smaller DICs (better fits) than the models that used the smaller administrative units (administrative 3 units).

| country | model | Admin 1: DIC (SD) | Admin 2: DIC (SD) | Admin 3: DIC (SD) |
| --- | --- | --- | --- | --- |
| Namibia | Basic | 1.68E+06 (460.0) | 6.12E+06 (2.7) | -- |
|  | Regional | -- | 4.61E+06 (4.0) | -- |
|  | Urbanicity | 9.23E+05 (1.9E+03) | 4.56E+06 (7.5) | -- |
|  | Regional- Urban | -- | 3.62E+06 (84.4) | -- |
| Kenya | Basic | 9.85E+07 (6.1E+05) | 3.48E+08 (3.8) | -- |
|  | Regional | -- | 3.40E+08 (135.0) | -- |
|  | Urbanicity | 6.11E+07 (4.8E+05) | 2.53E+08 (7.8) | -- |
|  | Regional- Urban | -- | 2.43E+08 (618.8) | -- |
| Burkina Faso | Basic | 1.01E+05 (215.1) | 2.67E+05 (3.1) | 1.07E+06 (2.8) |
|  | Regional | -- | 2.52E+05 (4.1) | 1.02E+06 (3.6) |
|  | Urbanicity | 5.86E+04 (1.9E+03) | 2.05E+05 (31.0) | 9.75E+05 (5.1) |
|  | Regional- Urban | -- | 1.93E+05 (18.4) | 9.36E+05 (8.5) |
| Zambia | Basic | 2.43E+05 (2.71E+03) | 2.40E+06 (2.9) | -- |
|  | Regional | -- | 2.08E+06 (4.2) | -- |
|  | Urbanicity | 2.42E+05 (2.54E+03) | 2.38E+06 (10.3) | -- |
|  | Regional- Urban | -- | 2.01E+06 (25.5) | -- |

**Supplementary file 2C.** Model fits ranked by Deviance Information Criterion (DIC) for each country. Although the different definitions of urbanicity impacted the distribution of urbanicity trip types, the overall ranking of the model fits was not affected. Generally, the models that used the lower urban threshold (10% urban grid cells) had larger DICs (worse fits) than the models that used the higher urban threshold (50% urban grid cells); however, the opposite was observed for Zambia. The gravity models with the power distance kernel were used here.

|  | Namibia | | Kenya | | Burkina Faso | | Zambia | |
| --- | --- | --- | --- | --- | --- | --- | --- | --- |
| rank | Model | DIC | Model | DIC | Model | DIC | Model | DIC |
| 1 | Regional-Urbanicity | Urb_50_: 3.6E+06  Urb_10_: 4.0E+06 | Regional-Urbanicity | Urb_50_: 2.4E+08  Urb_10_: 3.3E+08 | Regional-Urbanicity | Urb_50_:1.9E+05  Urb_10_:1.9E+05 | Regional-Urbanicity | Urb_50_: 2.0E+06  Urb_10_: 1.7E+06 |
| 2 | Urbanicity | Urb_50_: 4.6E+06  Urb_10_: 5.5E+06 | Urbanicity | Urb_50_: 2.5E+08  Urb_10_: 3.4E+08 | Urbanicity | Urb_50_:2.1E+05  Urb_10_:2.1E+05 | Urbanicity | Urb_50_: 2.4E+06  Urb_10_: 2.1E+06 |
| 3 | Regional | 4.6E+06 | Regional | 3.4E+08 | Regional | 2.5E+05 | Regional | 2.1E+06 |
| 4 | Basic | 6.1E+06 | Basic | 3.5E+08 | Basic | 2.7E+05 | Basic | 2.4E+06 |
| 5 | Radiation | 8.7E+06 | Radiation | 4.3E+08 | Radiation | 5.2E+06 | Radiation | 4.3E+06 |
