# Supplementary material for: Characterizing human mobility patterns in rural settings of sub-Saharan Africa
Source: eLife. 2021 Sep 17;10:e68441. doi: 10.7554/eLife.68441 (PMC8448534; doi:10.7554/eLife.68441)
Supplement: Supplementary file 3. — A. For each trip type and country, the model was reported that estimated the highest proportion of trips with estimated trip counts that fell within ±10%the observed trips (% trips). In situations where the proportion of trips of estimated by two models differed by less than 1%, both models were included. The distance kernel used is indicated by exp (exponential) or pwr (power). See B and C for the trip proportions for all models. B. For each trip type in each country, the percentage of estimated trips that fell within the selected interval of ±10% of the observed trip count. (Power distance kernel used in gravity models) C. For each trip type in each country, the percentage of estimated trips that fell within the selected interval of ±10% of the observed trip count. (Exponential distance kernel used in gravity models). [file elife-68441-supp3.docx]

**Supplementary file 3A.** For each trip type and country, the model was reported that estimated the highest proportion of trips with estimated trip counts within ± 10% of the observed trips. In situations where the proportion of trips of estimated by two models differed by less than 0.5%, both models were included. The distance kernel used is indicated by exp (exponential) or pwr (power). See **Supplementary File 3B-C** for the trip proportions for all models.

|  | **Namibia** | | **Kenya** | | | **Burkina Faso** | | **Zambia** | |
| --- | --- | --- | --- | --- | --- | --- | --- | --- | --- |
|  | Most accurate model | % trips | Most accurate model | % trips | Most accurate model | | % trips | Most accurate model | % trips |
| **Rural - Rural** | Regional-exp  Regional-Urbanicity-exp | 4.4  4.1 | Radiation | 6.4 | Regional-Urbanicity-exp | | 16.3 | Urbanicity - exp | 26.9 |
| **Rural - Urban** | Basic-pwr | 8.1 | Radiation  Urbanicity-pwr | 5.2  5.2 | Regional-Urbanicity-pwr  Urbanicity-pwr  Regional-pwr | | 11.4  11.4  11.4 | Basic – exp | 13.1 |
| **Urban - Rural** | Regional-Urbanicity-exp | 8.1 | Urbanicity-pwr  Regional-urbanicity-pwr | 4.4  4.3 | Regional-Urbanicity-pwr  Urbanicity-pwr | | 13.6  13.6 | Basic – exp | 13.1 |
| **Urban - Urban** | Regional-Urbanicity-pwr | 20.0 | Radiation | 4.1 | -- | | -- | Regional-pwr | 16.7 |
| **Intra-regional** | Regional-Urbanicity-pwr  Regional-Urbanicity-exp | 6.9  6.7 | Radiation  Regional-urbanicity-exp | 4.9  4.7 | Regional-pwr | | 9.2 | Regional-pwr | 4.2 |
| **Inter-regional** | Regional-exp  Regional-Urbanicity-exp | 4.7  4.3 | Radiation  Urbanicity-pwr | 4.4  4.3 | Regional-Urbanicity-exp | | 16.8 | Urbanicity - exp | 27.9 |
| **Intra:R-R** | Regional-Urbanicity-exp | 5.6 | Radiation | 6.9 | Regional-pwr | | 9.2 | Regional-exp | 3.9 |
| **Intra:R-U** | Regional-Urbanicity-pwr | 29.6 | Regional-Urbanicity-exp | 4.0 | -- | | -- | Regional-pwr | 11.1 |
| **Intra:U-R** | Regional-Urbanicity-pwr | 33.3 | Regional-Urbanicity-exp  Regional-pwr | 3.3  3.3 | -- | | -- | Regional-pwr | 11.1 |
| **Intra:U-U** | Regional-Urbanicity-pwr | 100 | Regional-Urbanicity-exp | 7.6 | **--** | | -- | -- | -- |
| **Inter:R-R** | Regional-exp  Regional-Urbanicity-exp  Regional-Model -pwr | 4.4  4.0  4.0 | Regional-Urbanicity-pwr | 5.8 | Regional-Urbanicity-exp | | 17.2 | Urbanicity - exp | 29.4 |
| **Inter:R-U** | Basic-pwr | 8.6 | Radiation | 4.9 | Urbanicity-exp | | 13.6 | Basic – exp | 13.6 |
| **Inter:U-R** | Regional-Urbanicity-pwr  Basic-pwr  Regional-exp  Regional-Urbanicity-exp | 6.8  6.8  6.8  6.4 | Urbanicity-pwr  Regional-Urbanicity-pwr  Radiation | 4.1  4.0  3.9 | Regional-Urbanicity-pwr  Urbanicity-pwr | | 13.6  13.6 | Basic – exp | 13.6 |
| **Inter:U-U** | Regional-exp | 17.9 | Urbanicity-pwr  Radiation | 4.9  4.4 | -- | | -- | Regional-pwr | 16.7 |

**Supplementary file 3B.** For each trip type in each country, the percentage of estimated trips that fell within ± 10% of the observed trip counts. (Power distance kernel used in gravity models)

|  | Basic | Urbanicity | Regional | Regional-Urbanicity | Radiation |
| --- | --- | --- | --- | --- | --- |
| Burkina Faso |  |  |  |  |  |
| Rural - Rural | 0.79 | 1.59 | 1.48 | 2.17 | 2.11 |
| Rural - Urban | 9.09 | 11.36 | 11.36 | 11.36 | 2.27 |
| Urban - Rural | 4.55 | 13.64 | 9.09 | 13.64 | 4.55 |
| Urban - Urban | NaN | NaN | NaN | NaN | NaN |
| Intra-regional | 1.54 | 2.31 | 9.23 | 8.46 | 6.92 |
| Inter-regional | 1.03 | 2.05 | 1.35 | 2.22 | 1.84 |
| Intra:R-R | 1.54 | 2.31 | 9.23 | 8.46 | 6.92 |
| Inter:R-R | 0.74 | 1.53 | 0.91 | 1.7 | 1.76 |
| Intra:R-U | NaN | NaN | NaN | NaN | NaN |
| Inter:R-U | 9.09 | 11.36 | 11.36 | 11.36 | 2.27 |
| Intra:U-R | NaN | NaN | NaN | NaN | NaN |
| Inter:U-R | 4.55 | 13.64 | 9.09 | 13.64 | 4.55 |
| Intra:U-U | NaN | NaN | NaN | NaN | NaN |
| Inter:U-U | NaN | NaN | NaN | NaN | NaN |
| Kenya |  |  |  |  |  |
| Rural - Rural | 2.53 | 4.14 | 2.99 | 4.25 | 6.44 |
| Rural - Urban | 1.54 | 5.21 | 3.08 | 4.1 | 5.21 |
| Urban - Rural | 1.45 | 4.36 | 2.22 | 4.27 | 2.56 |
| Urban - Urban | 3.1 | 3.24 | 3.04 | 3.04 | 4.12 |
| Intra-regional | 3.51 | 3.51 | 3.51 | 3.24 | 4.86 |
| Inter-regional | 1.95 | 4.3 | 2.71 | 3.95 | 4.35 |
| Intra:R-R | 2.3 | 3.45 | 1.72 | 2.87 | 6.9 |
| Inter:R-R | 2.58 | 4.64 | 2.41 | 5.84 | 3.95 |
| Intra:R-U | 1.97 | 1.97 | 2.63 | 1.32 | 2.63 |
| Inter:R-U | 1.61 | 3.51 | 3.31 | 4.12 | 4.92 |
| Intra:U-R | 2.63 | 1.97 | 3.29 | 1.32 | 2.63 |
| Inter:U-R | 1.61 | 4.12 | 2.61 | 4.02 | 3.92 |
| Intra:U-U | 5.73 | 5.34 | 5.34 | 5.73 | 6.11 |
| Inter:U-U | 2.18 | 4.86 | 2.47 | 2.98 | 4.43 |
| Namibia |  |  |  |  |  |
| Rural - Rural | 2.57 | 2.89 | 3.91 | 3.59 | 3.87 |
| Rural - Urban | 8.14 | 6.06 | 5.11 | 6.63 | 4.55 |
| Urban - Rural | 6.44 | 5.49 | 4.92 | 8.14 | 2.84 |
| Urban - Urban | 6.67 | 6.67 | 0 | 20 | 3.33 |
| Intra-regional | 3.44 | 5.39 | 3.74 | 6.89 | 3.89 |
| Inter-regional | 3.13 | 3.07 | 4.05 | 3.88 | 3.84 |
| Intra:R-R | 3.76 | 4.9 | 3.1 | 4.41 | 3.76 |
| Inter:R-R | 2.47 | 2.71 | 3.98 | 3.52 | 3.88 |
| Intra:R-U | 0 | 11.11 | 11.11 | 29.63 | 7.41 |
| Inter:R-U | 8.58 | 5.79 | 4.79 | 5.39 | 4.39 |
| Intra:U-R | 0 | 11.11 | 11.11 | 33.33 | 3.7 |
| Inter:U-R | 6.79 | 5.19 | 4.59 | 6.79 | 2.79 |
| Intra:U-U | 0 | 0 | 0 | 100 | 0 |
| Inter:U-U | 7.14 | 7.14 | 0 | 14.29 | 3.57 |
| Zambia |  |  |  |  |  |
| Rural - Rural | 0.71 | 0.91 | 1.01 | 1.63 | 5.15 |
| Rural - Urban | 1.98 | 1.98 | 2.72 | 3.71 | 3.71 |
| Urban - Rural | 1.98 | 1.98 | 2.72 | 3.47 | 2.23 |
| Urban - Urban | 0 | 0 | 16.67 | 0 | 8.33 |
| Intra-regional | 2.29 | 3.44 | 2.86 | 2.86 | 2.77 |
| Inter-regional | 0.65 | 0.73 | 0.97 | 1.66 | 5.23 |
| Intra:R-R | 2.05 | 3.28 | 2.25 | 2.66 | 2.46 |
| Inter:R-R | 0.57 | 0.66 | 0.88 | 1.52 | 5.44 |
| Intra:R-U | 5.56 | 5.56 | 11.11 | 5.56 | 5.56 |
| Inter:R-U | 1.63 | 1.63 | 1.9 | 3.53 | 3.53 |
| Intra:U-R | 5.56 | 5.56 | 11.11 | 5.56 | 8.33 |
| Inter:U-R | 1.63 | 1.63 | 1.9 | 3.26 | 1.63 |
| Intra:U-U | NaN | NaN | NaN | NaN | NaN |
| Inter:U-U | 0 | 0 | 16.67 | 0 | 8.33 |

**Supplementary File 3C.**  For each trip type in each country, the percentage of estimated trips that fell within ± 10% of the observed trip counts. (Exponential distance kernel used in gravity models).

|  | Basic | Urbanicity | Regional | Regional-Urbanicity | Radiation |
| --- | --- | --- | --- | --- | --- |
| Burkina Faso |  |  |  |  |  |
| Rural - Rural | 4.28 | 15.43 | 3.44 | 16.28 | 2.11 |
| Rural - Urban | 6.82 | 13.64 | 4.55 | 9.09 | 2.27 |
| Urban - Rural | 4.55 | 11.36 | 2.27 | 6.82 | 4.55 |
| Urban - Urban | NaN | NaN | NaN | NaN | NaN |
| Intra-regional | 3.08 | 4.62 | 6.15 | 3.85 | 6.92 |
| Inter-regional | 4.43 | 16.05 | 3.24 | 16.76 | 1.84 |
| Intra:R-R | 3.08 | 4.62 | 6.15 | 3.85 | 6.92 |
| Inter:R-R | 4.37 | 16.23 | 3.23 | 17.2 | 1.76 |
| Intra:R-U | NaN | NaN | NaN | NaN | NaN |
| Inter:R-U | 6.82 | 13.64 | 4.55 | 9.09 | 2.27 |
| Intra:U-R | NaN | NaN | NaN | NaN | NaN |
| Inter:U-R | 4.55 | 11.36 | 2.27 | 6.82 | 4.55 |
| Intra:U-U | NaN | NaN | NaN | NaN | NaN |
| Inter:U-U | NaN | NaN | NaN | NaN | NaN |
| Kenya |  |  |  |  |  |
| Rural - Rural | 1.84 | 2.3 | 2.76 | 2.64 | 6.44 |
| Rural - Urban | 2.31 | 1.54 | 2.99 | 1.54 | 5.21 |
| Urban - Rural | 2.65 | 1.62 | 3.16 | 2.39 | 2.56 |
| Urban - Urban | 2.5 | 3.51 | 2.7 | 3.31 | 4.12 |
| Intra-regional | 1.62 | 2.84 | 3.51 | 4.73 | 4.86 |
| Inter-regional | 2.51 | 2.23 | 2.78 | 2.1 | 4.35 |
| Intra:R-R | 0.57 | 4.02 | 2.3 | 2.3 | 6.9 |
| Inter:R-R | 2.75 | 2.41 | 2.58 | 2.75 | 3.95 |
| Intra:R-U | 1.97 | 1.97 | 2.63 | 3.95 | 2.63 |
| Inter:R-U | 2.31 | 1.61 | 3.41 | 1.61 | 4.92 |
| Intra:U-R | 1.32 | 1.32 | 1.97 | 3.29 | 2.63 |
| Inter:U-R | 2.51 | 1.71 | 3.51 | 2.31 | 3.92 |
| Intra:U-U | 2.29 | 3.44 | 5.73 | 7.63 | 6.11 |
| Inter:U-U | 2.54 | 2.98 | 1.89 | 2.03 | 4.43 |
| Namibia |  |  |  |  |  |
| Rural - Rural | 3.4 | 3.37 | 4.38 | 4.13 | 3.87 |
| Rural - Urban | 5.11 | 4.17 | 7.01 | 6.63 | 4.55 |
| Urban - Rural | 4.55 | 2.84 | 6.82 | 7.01 | 2.84 |
| Urban - Urban | 0 | 10 | 16.67 | 10 | 3.33 |
| Intra-regional | 2.84 | 3.29 | 4.64 | 6.59 | 3.89 |
| Inter-regional | 3.62 | 3.42 | 4.73 | 4.3 | 3.84 |
| Intra:R-R | 2.94 | 2.94 | 4.41 | 5.56 | 3.76 |
| Inter:R-R | 3.44 | 3.41 | 4.37 | 4 | 3.88 |
| Intra:R-U | 0 | 7.41 | 7.41 | 18.52 | 7.41 |
| Inter:R-U | 5.39 | 3.99 | 6.99 | 5.99 | 4.39 |
| Intra:U-R | 3.7 | 7.41 | 7.41 | 18.52 | 3.7 |
| Inter:U-R | 4.59 | 2.59 | 6.79 | 6.39 | 2.79 |
| Intra:U-U | 0 | 0 | 0 | 0 | 0 |
| Inter:U-U | 0 | 10.71 | 17.86 | 10.71 | 3.57 |
| Zambia |  |  |  |  |  |
| Rural - Rural | 25.33 | 26.89 | 16.91 | 18.93 | 5.15 |
| Rural - Urban | 13.12 | 9.9 | 9.16 | 7.92 | 3.71 |
| Urban - Rural | 13.12 | 9.9 | 9.16 | 7.92 | 2.23 |
| Urban - Urban | 0 | 0 | 0 | 0 | 8.33 |
| Intra-regional | 3.63 | 3.63 | 4.2 | 3.24 | 2.77 |
| Inter-regional | 26.6 | 27.94 | 17.61 | 19.67 | 5.23 |
| Intra:R-R | 3.28 | 3.28 | 3.89 | 2.87 | 2.46 |
| Inter:R-R | 27.69 | 29.42 | 18.3 | 20.65 | 5.44 |
| Intra:R-U | 8.33 | 8.33 | 8.33 | 8.33 | 5.56 |
| Inter:R-U | 13.59 | 10.05 | 9.24 | 7.88 | 3.53 |
| Intra:U-R | 8.33 | 8.33 | 8.33 | 8.33 | 8.33 |
| Inter:U-R | 13.59 | 10.05 | 9.24 | 7.88 | 1.63 |
| Intra:U-U | NaN | NaN | NaN | NaN | NaN |
| Inter:U-U | 0 | 0 | 0 | 0 | 8.33 |
